# From neuro-immune command circuits to microbiota-mediated regulation in the gastrointestinal tumor microenvironment

**DOI:** 10.3389/fimmu.2026.1739357

**Published:** 2026-01-28

**Authors:** Yuhan Chen, Dong Tang

**Affiliations:** 1 Clinical Medical College, Yangzhou University, Yangzhou, China; 2 Department of General Surgery, Institute of General Surgery Northern Jiangsu People’s Hospital Affliated to Yangzhou University, Yangzhou, China; 3 Northern Jiangsu People’s Hospital, Yangzhou, China; 4 The Yangzhou Clinical Medical College of Xuzhou Medical University, Yangzhou, China

**Keywords:** cancer, enteric nervous system, gut microbiota, immunotherapy, neuroimmunity

## Abstract

The nervous system plays a profound role in human health and disease, particularly in regulating cancer development through immune system interactions. The enteric nervous system (ENS), often referred to as the “second brain,” comprises millions of neurons and glial cells specialized for the gastrointestinal tract. This system is intimately involved in the growth, infiltration, and metastasis of gastrointestinal tumors. Furthermore, the ENS establishes a bidirectional communication network with the central nervous system via the vagus nerve and spinal afferent nerves, mediating interactions between gut microbiota, the immune system, and the nervous system. Emerging fields like “neuro-immuno-oncology” have introduced neuroimmunomodulatory drugs into clinical practice, but most research focuses on intestinal inflammation, leaving a gap in systematic understanding regarding gastrointestinal tumors. This review systematically summarizes the bidirectional regulatory mechanisms of neuro-immune interactions in gastrointestinal tumors and explores the interplay between nerves, immunity, and microbiota in the gastrointestinal tumor microenvironment. Its aim is to provide a new perspective for understanding the neuro-immune ecology of gastrointestinal tumors and to lay a theoretical foundation for developing cross-scale precision treatment strategies.

## 1 Introduction

The gastrointestinal tumor microenvironment (TME) is a dynamic ecosystem, encompassing tumor cells, nerve fibers, immune cells, and gut microbiota. Within this ecosystem, neuro-immune interactions propel tumor progression and immune evasion through a multifaceted signaling network (1, 2). Both the central nervous system (CNS) and peripheral nerves finely tune the phenotype and function of immune cells through neurotransmitters, neuropeptides, and neurotrophic factors, forming a complex spatiotemporal interaction pattern. The ENS stands as the pivotal hub in this network, directly modulating immune cell function via neurotransmitter and neuropeptide release. Composed of the myenteric and submucosal plexuses, the ENS independently regulates gastrointestinal motility and secretion through local neural circuits (3). Recent research reveals that the gastrointestinal nervous system orchestrates digestion, secretion, immunity, and metabolic functions through the autonomous regulation of the ENS and its integration with the autonomic nervous system (ANS) and central nervous system (CNS) (4, 5). Besides directly regulating tumor-infiltrating immune cell function, the ENS engages in dynamic interactions with the CNS via the vagus nerve-spleen axis, mediating remote regulation of gut microbiota metabolites on systemic immune responses. Gut microbiota, as the linchpin of the gut-brain axis, shapes distal immune responses through metabolic reprogramming and neural signaling (6). However, current research often focuses on single pathways or cell types, and there is still a lack of systematic understanding of the dynamic spatiotemporal characteristics and cross-organ regulatory networks of neuro-immune interactions. Based on the unique characteristics of the gastrointestinal tract within the neuroimmune framework, this review provides an in-depth analysis of the bidirectional crosstalk between the nervous and immune systems, as well as the involvement of the gut microbiota. It specifically summarizes the regulatory mechanisms of common gut microbiota in the tumor microenvironment of gastrointestinal cancers, elucidates the spatiotemporal dynamics and specificity of gastrointestinal tumors from multiple perspectives, and integrates current clinical challenges to offer novel insights and a theoretical foundation for cross-scale precision therapy in gastrointestinal oncology.

## 2 Neural command and immune counterattack: the dual-track regulatory network of the gastrointestinal tumor microenvironment

The development and progression of gastrointestinal tumors are closely associated with dynamic interactions with the nervous system, a process involving multi-level regulation by the central nervous system (CNS), peripheral nervous system (PNS), and enteric nervous system (ENS). The bidirectional regulation of the neuro-immune axis is a central feature of the tumor microenvironment (TME) in gastrointestinal cancers. We will next dissect the gastrointestinal TME from the perspective of two systems with competing effects. On one hand, the nervous system releases neurotransmitters, neuropeptides, and neurotrophic factors, which modulate immune cell behavior and accelerate tumor progression. For instance, β-adrenergic receptors (ADRBs) are upregulated in gastrointestinal tumors, where sympathetic activation enhances epinephrine (Epi) and norepinephrine (NE) secretion within the TME (7). NE engages β2-adrenergic receptors (β2-AR) on tumor-associated macrophages (TAMs), activating the cAMP/PKA axis, inducing M2 polarization and secretion of interleukin-10 (IL-10) and TGF-β, thereby promoting tumor cell proliferation and suppressing apoptosis (8). On the other hand, immune cells, including TAMs, myeloid-derived suppressor cells (MDSCs), and group 3 innate lymphoid cells (ILC3s), possess “defense programs” that can either resist or, when hijacked, reinforce neural commands.

### 2.1 Neural molecular signals that reprogram the TME

#### 2.1.1 NGF

Nerve growth factor (NGF), a pivotal neurotrophic factor, operates through a circulatory mechanism involving multiple cellular sources, receptor-dependent signaling, and the reshaping of the immune microenvironment. The cholinergic-NGF signaling axis constitutes the molecular basis for neuro-immune interactions in gastrointestinal tumors. Specifically, acetylcholine (ACh) stimulates NGF expression via cholinergic signaling, while NGF promotes the growth of cholinergic nerve axons through its high-affinity receptor, TrkA (9, 10). NGF activates diverse signaling pathways by binding to both the high-affinity receptor TrkA and the low-affinity receptor p75NTR. The TrkA-mediated PI3K/Akt/mTOR pathway promotes neuronal survival, axon growth, and synaptic plasticity, with reduced NGF levels correlating with diminished axon outgrowth (11).

Conversely, the p75NTR receptor can trigger apoptosis or inhibit pathological neural hyperplasia by activating the RhoA/ROCK and JNK/c-Jun signaling cascades. Within the tumor microenvironment, the NGF-TrkA signaling axis drives tumor-associated neurogenesis (TAN). NGF secreted by tumor cells attracts infiltrating sensory nerve axons, which release calcitonin gene-related peptide (CGRP) to activate the RAMP1 receptor on tumor cells (12). This activation initiates PI3K-Akt/CaMK pathway-mediated phosphorylation of Rb protein and the release of E2F transcription factors, establishing a self-sustaining NGF-CGRP positive feedback loop. This mechanism also reshapes the immunosuppressive microenvironment by significantly boosting the IL-6 secretory capacity of cancer-associated fibroblasts (CAFs), thereby inhibiting cytotoxic T cell function (13, 14).

Furthermore, NGF promotes immune evasion by modulating immune cell functions: TrkA activation on macrophages drives M2 phenotype polarization and the secretion of IL-10 and TGF-β. NGF can also upregulate programmed cell death protein (PD-1) expression in CD8+ T cells via the β2-AR/cAMP signaling pathway, which inhibits mitochondrial oxidative phosphorylation and contributes to T cell exhaustion (15).

A separate investigation into apoptotic mechanisms revealed that NGF not only contributes to tumor progression but can also trigger neuronal apoptosis upon its deprivation. Interestingly, this apoptotic signal is transiently activated, permitting a window for neuronal recovery. This recovery depends on the expression of the anti-apoptotic protein Bcl-xL. Consequently, neurons exposed to apoptotic signals in the absence of NGF can restore survival via these transient signals, effectively undergoing a “reset” (16, 17). This finding implies that tumor cells may similarly exploit transient apoptotic signals for survival, providing a novel perspective for therapeutic intervention.

#### 2.1.2 BDNF

Within the stressful tumor microenvironment, matrix metalloproteinase 9 (MMP9) upregulation accelerates the maturation of brain-derived neurotrophic factor (BDNF) (18). BDNF demonstrates potential diagnostic value, given its abnormal expression in gastrointestinal tumors (19). Lactate produced by these tumor cells via aerobic glycolysis is transported into CAFs through monocarboxylate transporter 1 (MCT1). This lactate influx acidifies the intracellular environment of CAFs, which activates the NF-κB signaling pathway and stimulates substantial BDNF secretion. The CAF-derived BDNF then nourishes tumor cells, persistently activating their surface TrkB signaling to establish a positive feedback loop (20). This circuit enhances tumor cell invasiveness and metastatic potential while remodeling the local immunosuppressive microenvironment into a chemotherapy-resistant niche. These findings offer a mechanistic explanation for the low chemotherapy sensitivity observed in some gastrointestinal tumor patients (21). To verify the functional necessity of BDNF in drug resistance, researchers used CRISPR/Cas9 to generate a BDNF-knockout gastrointestinal tumor cell model for ex vivo drug sensitivity assays. The knockout cells exhibited significantly increased sensitivity to chemotherapeutic agents like anlotinib and showed elevated drug-induced accumulation of reactive oxygen species (ROS). This directly confirmed that BDNF antagonizes chemotherapy-induced cytotoxicity by regulating intracellular redox homeostasis and apoptotic signaling pathways (22). Another study demonstrated that the long non-coding RNA BDNF-AS recruits WDR5 to regulate FBXW7 transcription, thereby inhibiting FBXW7-mediated ubiquitination and degradation of VDAC3, maintaining mitochondrial membrane stability, and ultimately counteracting ferroptosis (23). This finding expands the known mechanisms by which BDNF and its regulatory network confer resistance to tumor cell death. However, key issues remain: current research focuses predominantly on the singular regulatory effects of the BDNF-TrkB pathway, while potential synergistic or antagonistic interactions between CAF-derived BDNF and other cells in the TME have not been systematically investigated. Although BDNF-knockout experiments confirm its necessity in drug resistance, clinical trials of small-molecule TrkB inhibitors in gastrointestinal tumors have shown limited efficacy. Despite these limitations, BDNF and its regulatory network represent potential targets for precision therapy in gastrointestinal tumors, providing a clear direction for subsequent mechanistic studies and drug development.

#### 2.1.3 Netrin-1

Beyond neurotrophic factors, neuroimmunity involves numerous molecular signals. Netrin-1, a key axon guidance molecule, functions as a central regulator of neuroimmune crosstalk. It modulates both the immunosuppressive activity of MDSCs and the homeostasis of cancer stem cells (CSCs). In colorectal cancer, tumor-secreted Netrin-1 binds to the adenosine A2B receptor (A2BR) on MDSCs. This binding activates the cAMP/PKA pathway and induces cAMP-response element binding protein (CREB) phosphorylation, which potently enhances MDSC-mediated immunosuppression. Consequently, MDSCs inhibit CD8+ T cell proliferation and promote regulatory T cell (Treg) differentiation, thereby shaping an immunosuppressive tumor microenvironment (24, 25). Conversely, IL-4 and IL-13 secreted by TAMs upregulate neuronal Netrin-1 expression via STAT6 signaling, establishing a positive feedback loop that accelerates nerve fiber hyperplasia and tumor innervation. Furthermore, deleted in colorectal carcinoma (DCC) is a functional dependence receptor (DepR). The interaction between DCC and Netrin-1 can induce the phosphorylation of FAK, Rac1, and Cdc42, which plays a crucial role in axonal growth (26). Studies on oligodendrocyte death following brain injury have found that targeted inhibition of Netrin-1 expression or blockade of its interaction with DCC can restore the apoptotic capacity of tumor cells. This provides a molecular basis for developing precise therapeutic strategies for coloectal cancer (CRC) (27).

#### 2.1.4 Adenosine

Adenosine (ADO), a key neuromodulator, is predominantly produced via the CD39-CD73 pathway, where extracellular ATP is hydrolyzed to AMP by CD39 and subsequently dephosphorylated to ADO by CD73. This enzymatic cascade is significantly upregulated in tumor cells, CAFs, and MDSCs, particularly under the hypoxic and oxidative stress conditions governed by the HIF-1α/NF-κB pathway (28). Within the TME, ADO influences tumor progression through diverse cellular and signaling mechanisms. ADO released from tumor-associated neurons, for instance, activates the A2B receptor on astrocytes, inducing metabolic reprogramming and increased lactate secretion that fuels aerobic glycolysis in cancer cells. Conversely, ADO binding to the A2A receptor on CD8^+^ T cells triggers the cAMP–PKA pathway, which directly inhibits T cell receptor (TCR) signaling and diminishes the release of interferon-γ (IFN-γ) and granzyme B, thereby attenuating antitumor immunity (29, 30). Preclinical evidence further indicates that A2A receptor overexpression in gastric and hepatocellular carcinomas promotes epithelial–mesenchymal transition (EMT) via the PI3K–AKT–mTOR axis, enhancing tumor invasion and metastasis. Meanwhile, ADO secreted by CAFs upregulates their own CD73 expression in a feedforward manner, creating a self-amplifying “ADO–CD73–ADO” circuit that exacerbates immunosuppression in the TME and contributes to chemotherapy resistance (31, 32). Current clinical research, however, is still predominantly focused on liver cancer and pancreatic ductal adenocarcinoma (PDAC), with a paucity of high-quality clinical data for other gastrointestinal malignancies. Most insights also remain heavily dependent on murine models. While molecules like CD39 and CD73 are well-studied, other critical regulators of ADO metabolism within the TME are not fully defined. Consequently, the regulatory landscape of ADO metabolism remains incomplete, hindering the development of precise therapeutic approaches (33). Given the reported toxicity of agents such as A2A receptor inhibitors, more tumor-selective and precise treatment strategies are therefore needed.

**Figure 1:**
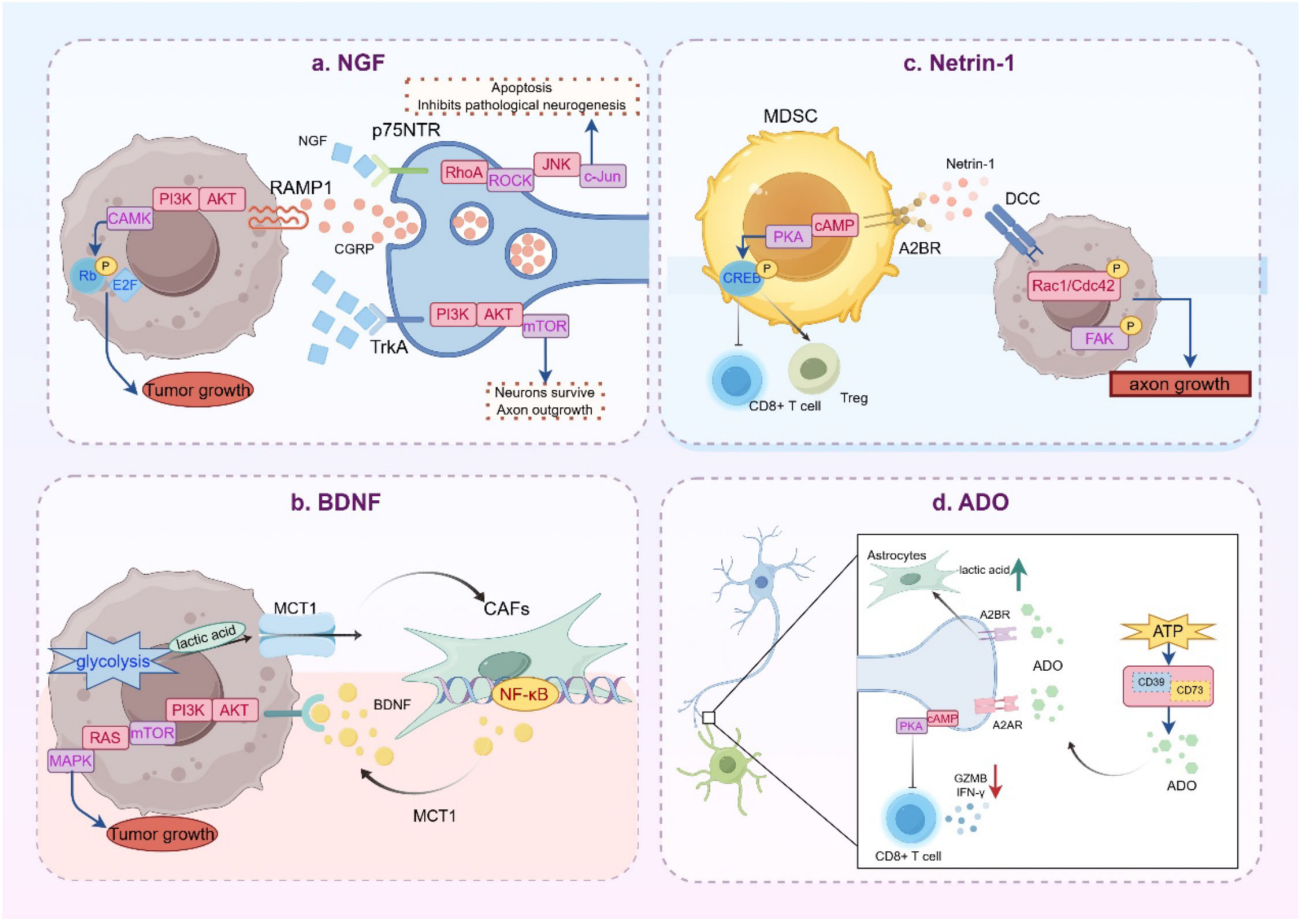
Molecular signaling in gastrointestinal tumors. **(A)** NGF specifically binds to the high-affinity TrkA receptor, activating the PI3K/Akt/mTOR pathway, and works together with the low-affinity receptor p75NTR to exert immune functions. Tumor cells recruit sensory nerves to release CGRP, which triggers the PI3K-Akt/CaMK pathway via the RAMP1 receptor, forming a positive feedback loop known as the “NGF-CGRP” circuit. **(B)** BDNF binds to the TrkB receptor, activating both the PI3K/AKT/mTOR and RAS/MAPK pathways, thereby enhancing tumor survival. Lactate activates NF-κB-dependent BDNF secretion through CAFs, creating a drug-resistant niche. **(C)** Netrin-1 binds to the A2BR receptor on MDSCs, activating the cAMP/PKA/CREB pathway and enhancing immunosuppressive functions. It also induces the phosphorylation of FAK, Rac1 and Cdc42 to promote axon growth via the DCC receptor. **(D)** The CD39-CD73 cascade generates ADO, which inhibits CD8+ T cell function via the adenosine A2A receptor (A2AR) and activates PI3K-AKT-mTOR to induce epithelial-mesenchymal transition (EMT).

In conclusion, the bidirectional regulation of neuroimmunity in gastrointestinal tumors is driven by a diverse and intertwined molecular signaling network. These signals form multi-level interactions through transmembrane receptors, epigenetic modifications, and metabolic reprogramming. Together, they shape an immunosuppressive, neuroplastic, and metabolically adaptive tumor ecology (**Figure 1**). These signals also represent potent molecular targets for the treatment of gastrointestinal tumors, offering new directions for future therapies.

### 2.2 Immune cell programs that shape tumor fate

#### 2.2.1 Glial cell

**Figure 2:**
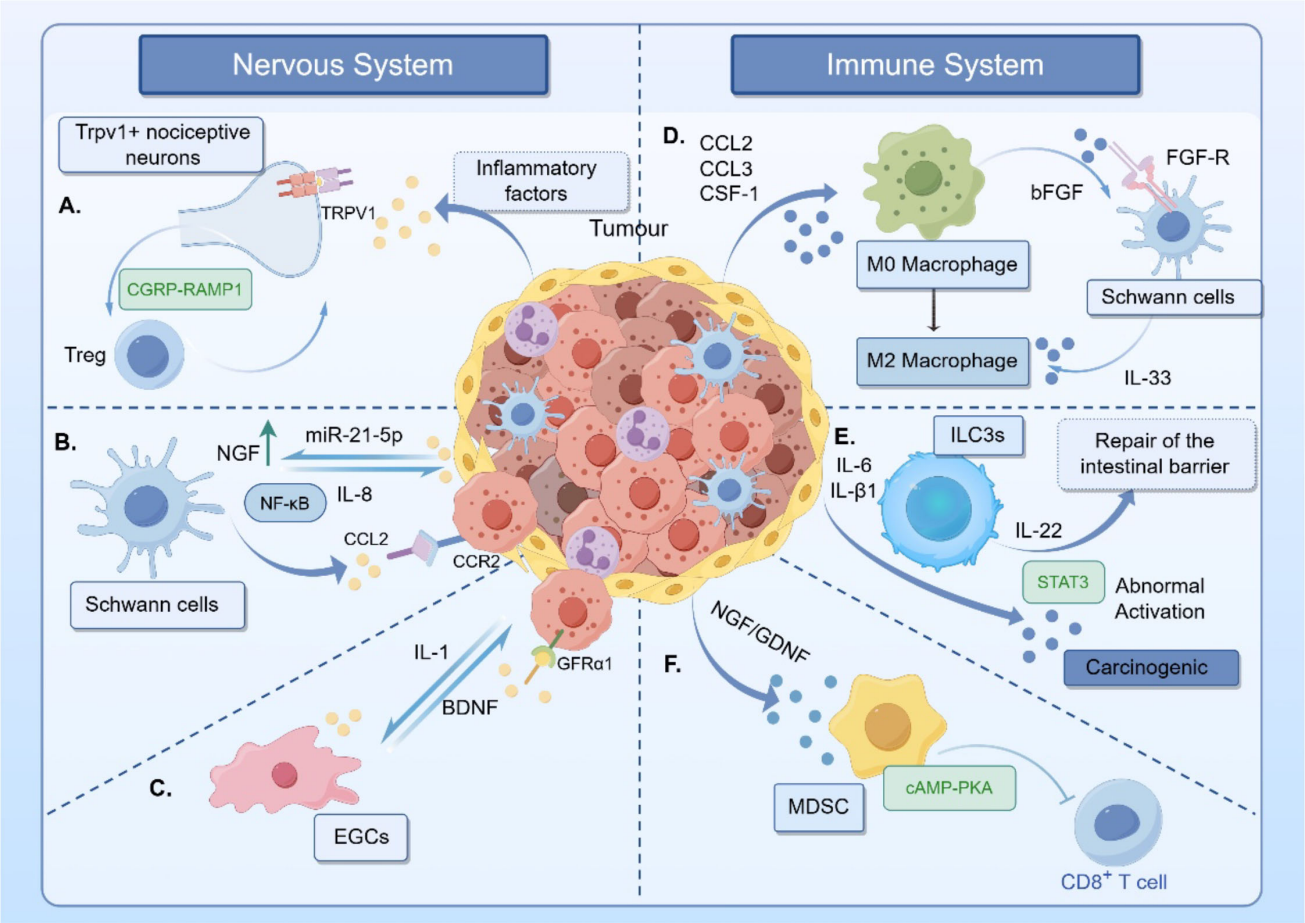
Interactions between immune cells and the intestinal tumor microenvironment. **(A)** Tumor cells secrete inflammatory factors to activate transient receptor potential vanilloid 1 (TRPV1) neurons, thereby promoting Treg function via the CGRP-RAMP1 axis; Tregs also promote neuronal growth. **(B)** Tumor cells upregulate NGF expression via exosomal miR-21-5p, creating a pro-tumor environment. NGF also stimulates Schwann cell proliferation, accelerating tumor infiltration and metastasis. Additionally, Schwann cells activate the NF-κB pathway, inducing IL-8 secretion and promoting cancer progression. **(C)** Tumor cells secrete IL-1 to promote EGCs, which secrete GDNF to enhance cancer cell invasiveness by binding to the GFRα1 receptor. **(D)** TAMs secrete bFGF, driving Schwann cells to secrete IL-33, thereby inducing macrophage M2 polarization. **(E)** Tumor cells hijack IL-22 secreted by ILC3s through factors like IL-6, leading to abnormal STAT3 activation and promoting tumor growth. **(F)** Tumor-secreted factors such as NGF and GDNF enhance the inhibitory effects of MDSCs, which suppress immune cell function via cAMP-PKA signaling.

Recent research has further clarified the close association between gut nerves and immune cells (**Figure 2**). A chemogenetic screening study revealed that Trpv1+ nociceptive neurons activate intestinal Treg cell expression via the CGRP-RAMP1 signaling pathway, while Treg cells conversely promote the regeneration of these neurons (34). Perineural invasion (PNI) is a pivotal process in tumor infiltration and metastasis of gastrointestinal cancers. Zhao et al. identified that Schwann cells, the primary glial cells of the PNS, contribute to regulating NGF expression. Specifically, CRC cells downregulate von Hippel-Lindau (VHL) protein in Schwann cells via exosomal miR-21-5p. The downregulation of VHL protein leads to the stabilization of hypoxia-inducible factor 1α (HIF-1α), which subsequently upregulates the expression level of NGF, thereby forming a positive feedback loop that promotes cancer progression. In turn, NGF further stimulates the proliferation of Schwann cells, accelerating tumor infiltration and metastasis (35, 36). NGF then further stimulates Schwann cell proliferation, accelerating tumor infiltration and metastasis. These findings indicate that targeting NGF or exosomal miR-21-5p may have therapeutic potential. Within the tumor-glial niche, Schwann cells in the CRC microenvironment can activate the NF-κB pathway, inducing IL-8 secretion and creating a vicious cycle that advances cancer (37). Furthermore, TAMs can drive Schwann cells to secrete IL-33 via the secretion of basic fibroblast growth factor (bFGF), thereby promoting macrophage M2 polarization and facilitating neural invasion (38). Schwann cells also secrete CCL2 (monocyte chemoattractant protein-1), which binds to CCR2 receptors on cancer cells. This interaction promotes cancer cell migration along nerves and recruits immune cells, such as monocytes and memory T cells, to the tumor microenvironment, forming a pro-inflammatory network that fuels cancer growth. Blocking the CCL2-CCR2 signal has been found to significantly inhibit PNI (13).

Enteric glial cells (EGCs), a distinct intestinal glial population, are integral to the enteric nervous system and modulate the gastrointestinal tumor immune microenvironment through phenotypic plasticity and secretory functions (39). Experimental evidence indicates that depleting EGCs can slow cancer progression. EGCs secrete glial cell-derived neurotrophic factor (GDNF), which enhances cancer cell invasiveness by binding to GFRα1 receptors (40). Depleting EGCs experimentally slows tumor progression. These cells release GDNF, which binds GFRα1 receptors to increase cancer cell invasiveness. EGCs also secrete NGF and BDNF, stimulating neuronal branching and tumor-nerve interactions to accelerate perineural invasion (41).

#### 2.2.2 Intrinsic lymphocytes

Type 3 innate lymphoid cells (ILC3s), which are enriched in the intestinal mucosa and regulate the intestinal barrier, operate via a dual-signal mode mediated by β2-adrenergic receptors (β2-AR) and vasoactive intestinal peptide receptor 2 (VIPR2) (42). Sympathetic nerve-derived norepinephrine activates IL-22 transcription through β2-AR. In mouse models, Wang et al. demonstrated *in vitro* that IL-22 facilitates epithelial repair via signal transducer and activator of transcription 3 (STAT3) pathway (43). Notably, STAT3, a pathway commonly involved in CRC development, has been shown to act as a tumor growth factor in certain contexts, driving DNA damage and carcinogenesis under chronic inflammation (44). On the other hand, ILC3 directly senses intestinal neuronal signals through neuropeptide receptors such as VIPR2 and NMUR1. Experiments have shown that VIP, secreted by intestinal neurons, induces SHP-1 phosphorylation in CCR6+ ILC3 via VIPR2, inhibiting STAT3-mediated antimicrobial peptide expression. This promotes lipid absorption but elevates the risk of intestinal microbiota dysbiosis in obesity-related colorectal cancer models (45, 46). Further studies by Shao et al. revealed the key balancing role of the transcription factor FOXO1 in this process. Under homeostasis, FOXO1 promotes VIPR2 transcription while repressing expression of the adrenergic receptor ADRA2A, enabling ILC3s to preferentially respond to neuronal VIP signals and sustain IL-22-dependent barrier function (47). Further studies by Shao et al. revealed the key balancing role of the transcription factor FOXO1 in this process. Under homeostasis, FOXO1 promotes VIPR2 transcription while repressing expression of the adrenergic receptor ADRA2A, enabling ILC3s to preferentially respond to neuronal VIP signals and sustain IL-22-dependent barrier function (47). During chronic stress or within the tumor microenvironment, however, sustained sympathetic activation amplifies cAMP-PKA signaling, leading to FOXO1 phosphorylation and degradation. This shifts ILC3s toward an ADRA2A-dominated state, attenuates IL-22 secretion, and heightens ILC3 sensitivity to norepinephrine, thereby exacerbating cAMP accumulation and establishing a positive feedback loop.

ILC3s also respond to tissue damage via the PGE2-EP2 axis (48). Following IL-1β activation, autocrine PGE2 signaling in ILC3 induces the expression of pro-heparin-binding EGF-like growth factor (pro-HB-EGF), which is then cleaved to release soluble HB-EGF. This soluble factor inhibits TNF-induced epithelial cell apoptosis through epidermal growth factor receptor (EGFR) signaling, preserving barrier integrity. However, in chronic inflammation, the depletion of ILC3 leads to decreased HB-EGF levels, exacerbating TNF-mediated intestinal epithelial damage, thus creating a vicious cycle linking inflammation, barrier disruption, and tumor progression.

ILC3 also produces inflammatory factors, including IL-17A and IL-17F (49). In maintaining ILC3 homeostasis, IL-17D regulates ILC3’s functional stability through CD93 (50). Recent studies have also discovered that intestinal GABAergic neurons can stabilize intestinal homeostasis by inhibiting the release of the inflammatory factor IL-17A through their secreted neurotransmitters (51). Another study reported a positive correlation between the transmembrane protein neuropilin-1 (NRP1) and ILC3s, suggesting NRP1 as a potential target for barrier maintenance through NF-κB–mediated regulation of IL-17A production (52). In summary, ILC3s play a dual role in gastrointestinal tumors by participating in a complex neuroimmune network and influencing intestinal microbiota. The gastrointestinal neuroimmune environment, one of the most neuronally dense sites, involves cellular mechanisms far more complex and diverse than those highlighted here. Deeper exploration of these mechanisms remains a promising direction for future research. The table below summarizes recent mechanistic studies on relevant immune cells (**Table 1**).

**Table 1:** Common pathways of gastrointestinal immune cells in neuroimmunity.

Immune cell type	Signaling pathways	Key targets/molecules	Function	Reference
Tumor-associated macrophages (TAMs)	β2-AR/cAMP-PKA pathway	β2-AR	NE drives M2 polarization via β2-AR, secretes IL-10, TGF-β, and inhibits CD8+ T cell activity.	(8)
	CGRP-RAMP1 axis	RAMP1 receptor	CGRP activates CAFs to secrete IL-6, enhances the M2 phenotype of TAMs, and promotes tumor growth.	(53)
	JAK2/STAT3 pathway	STAT3	TAMs can release IL6, activate the JAK2/STAT3 signaling pathway, and down-regulate the oncogene miR-506-3p.	(54)
Myeloid-derived suppressor cells (MDSCs)	Vagus-TFF2/CXCR4 axis	CXCR4 receptor	Vagal activation induces splenic TFF2 secretion and blocks PD-L1 and ARG1 expression in MDSCs.	(55)
	Sympathetic-β2-AR/cAMP pathway	β2-AR	NE promotes MDSCs expansion through β2-AR, inhibits T cell function, and accelerates tumor metastasis.	(56)
	Adenosine-A2B receptor pathway	A2BR	Adenosine in the tumor microenvironment activates cAMP-PKA signaling via A2B receptor and inhibits TCR signaling.	(24)
CD8 T cells	β2-AR/PD-1 signaling	PD-1 receptor	NE upregulates PD-1 expression through β2-AR, inhibits T cell toxicity, and induces T cell depletion.	(57)
	5-HT-TGM2/GAPDH axis	TGM2 enzyme、GAPDH	5-HT mediates GAPDH 5-HTylation via TGM2, enhances glycolytic metabolism, and supports T cell effector function.	(58)
Regulatory T cells (Tregs)	NPY-Y1 receptor signaling	Y1 receptor	ENS releases NPY to activate the MAPK pathway via Y1 receptor, promoting Tregs expansion and secretion of IL-10.	(59)
	Adenosine-A2A receptor pathway	A2A receptor	Adenosine in the tumor microenvironment activates cAMP-PKA signaling via A2A receptor and inhibits TCR signaling.	(29)
Type 3 congenital lymphocytes (ILC3)	VIP-VIPR2/STAT3 axis	VIPR2 receptor、STAT3	VIP inhibits STAT3-dependent antimicrobial peptide expression via VIPR2, leading to dysbiosis and promoting cancer.	(43)
	β2-AR/IL-22 pathway	β2-AR	NE drives IL-22 secretion from ILC3 via β2-AR, promoting epithelial repair or abnormal proliferation	(42)
Carcinoma-associated fibroblasts (CAFs)	CGRP-RAMP1/IL-6 axis	RAMP1 receptor、IL-6	CGRP activates CAFs to secrete IL-6, promoting TAMs M2 polarization and tumor cell proliferation.	(60, 61)
	BDNF-TrkB/EGFR pathway	TrkB receptor、EGFR	CAFs secreted BDNF to activate tumor cell EGFR signaling through TrkB, promoting invasion and metastasis.。	(20)
Intestinal glial cells (EGCs)	GDNF-GFRα1 axis	GFRα1 receptor	EGCs secreted GDNF to enhance gastric cancer cell invasiveness and promote perineural infiltration (PNI).	(41)
	NGF-TrkA pathway	TrkA receptor	EGCs secreted NGF to drive sensory nerve axon proliferation and accelerate tumor innervation.	(41)
Schwann cells	miR-21-5p/VHL-HIF-1α axis	VHL protein、HIF-1α	CRC exosome miR-21-5p downregulates VHL in Schwann cells, stabilizes HIF-1α and upregulates NGF to promote carcinogenesis.	(35)
	NF-κB/IL-8 pathway	NF-κB、IL-8	Schwann cells activate NF-κB to secrete IL-8, promoting cancer cell migration and angiogenesis.	(37)

## 3 The microbial modulator: gut microbiota rewires the neuro-immune circuit

**Figure 3:**
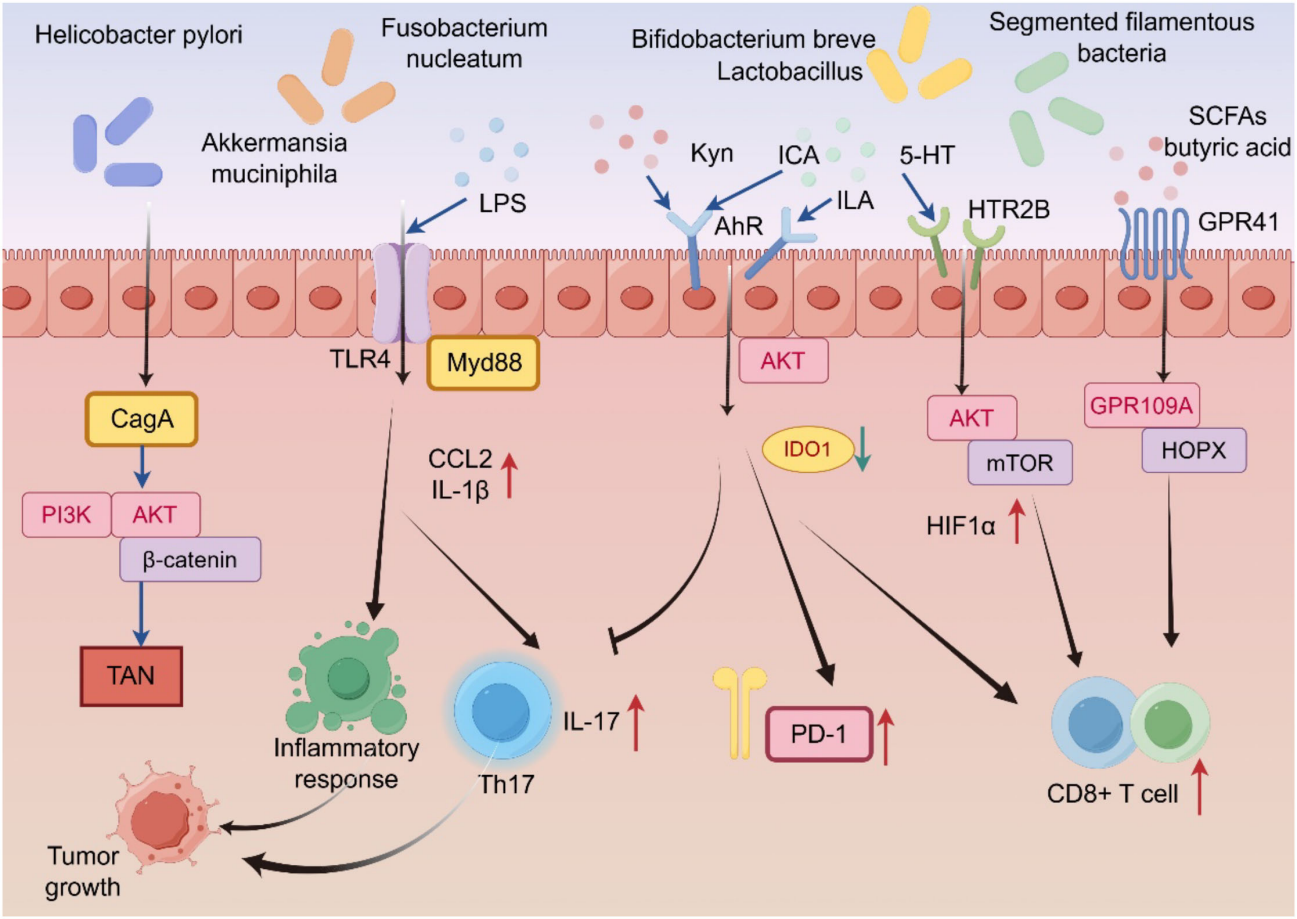
Mechanisms of action of gut microbiota and their metabolites in the gastrointestinal tract. Helicobacter pylori activates the PI3K-AKT-β-catenin signaling axis through the CagA virulence factor, driving TAN. Fn-secreted LPS promotes macrophage inflammatory responses via TLR4/Myd88. 5-HT activates the PI3K/mTOR pathway through the HTR2B receptor, facilitating tumor metabolic reprogramming and metastasis. SCFAs like butyrate inhibit PD-L1/IL-10 expression in TAMs via the GPR109A receptor, improving the immune microenvironment. Tryptophan-metabolizing bacteria activate the AhR-IDO1 pathway through kynurenine (Kyn), suppressing T cell function and forming an immunosuppressive microenvironment.

Although the preceding sections have described how intrinsic host neural circuits exert molecular command over immune cell fate, this neuroimmune hierarchy is neither fixed nor unidirectional. Instead, it is continuously remodeled by the dynamic consortium of gut microbiota—microorganisms that sense, intercept, and rewire host signaling pathways. Specific pathogens and microbial metabolites not only respond to tumor-associated inflammatory reactions; they also actively modulate neurotransmitter availability, neuropeptide secretion, and immune cell responses, thereby reconfiguring the balance of power within the tumor microenvironment (62, 63). The following section primarily introduces the mechanisms by which gut microbiota and their metabolites participate in the regulation of gastrointestinal tumors through the neuroimmune system (**Figure 3**).

### 3.1 Microbiota as a source of neuroactive and immunomodulatory metabolite

Gut microbiota-derived metabolites are pivotal regulators of the tumor microenvironment in gastrointestinal cancers. These metabolites often share significant structural or functional similarities with host-derived signaling molecules. Through such molecular mimicry, the microbiota can directly and precisely influence host cell functions without needing to colonize the tumor site. The survival and regeneration of enteric neurons, for example, depend on continuous microbial stimulation. Lipopolysaccharide (LPS), a component of the Gram-negative bacterial cell wall, binds to Toll-like receptor 4 (TLR4) on enteric neurons and glial cells to activate downstream survival pathways (64, 65). This effectively suppresses neuronal apoptosis and ensures neuronal survival. Meanwhile, TLR2 activation contributes to neurogenesis. Together, these mechanisms coordinately maintain ENS function (66). Under pathological conditions, however, such neuroprotective mechanisms can be subverted: studies in mouse models indicate that while exogenous LPS supplementation inhibits neuronal loss after chemical injury, it fails to restore gastrointestinal motility (67, 68). This suggests that microbiota-mediated regulation of the nervous system is complex and multi-layered.

Furthermore, the microbiota directly modulates the host’s neurochemical signaling network by synthesizing and transforming neurotransmitters. Levodopa (L-DOPA), a key therapeutic precursor in Parkinson’s disease, is also metabolized by the gut microbiota. Within the intestine, abundant bacterial tyrosine decarboxylase converts exogenous or endogenous L-DOPA into dopamine (DA). This microbial conversion significantly increases luminal dopamine levels, establishing the gut microbiota as the primary driver of intestinal dopaminergic signaling (69). Moreover, several bacterial species, including Staphylococcus aureus and Escherichia coli, are capable of directly synthesizing ACh. This microbially derived ACh can act directly on intestinal epithelial cells and enteric neurons, contributing to the maintenance of neural homeostasis (70). Similarly, enterochromaffin (EC) cells in the gastrointestinal tract represent the primary source of peripheral serotonin (5-hydroxytryptamine, 5-HT) and form synapse-like structures with sensory nerve endings to facilitate signal transmission (71).

In summary, the gut microbiota functions as a dynamic endocrine organ, continuously releasing bioactive molecules into the systemic circulation and the local tumor microenvironment. This process is jointly determined by microbial ecology and host diet, and it continuously shapes the transcriptional state, metabolic phenotype, and functional properties of neurons and immune cells. Subsequent sections will further explore how the overall metabolic output of the gut microbiota may skew toward either promoting or inhibiting tumor progression.

### 3.2 Pathobiont-induced reprogramming: the case of *H. pylori* and *F. nucleatum*

Within the intricate ecosystem of the gut microbiota, certain bacterial members with established pathogenicity actively disrupt the host’s neuro-immune signaling circuits during homeostasis, thereby reshaping the tumor microenvironment. The specific mechanisms of Helicobacter pylori and Fusobacterium nucleatum cereus are detailed below.

#### 3.2.1 Helicobacter pylori

Helicobacter pylori (Hp) infection serves as a critical driver of gastrointestinal carcinogenesis, reshaping the TME through intricate neuroimmune interactions (72). The progression from infection to gastric atrophy, intestinal metaplasia, and gastric adenocarcinoma (GAC) involves H. pylori-mediated regulation of bidirectional nervous and immune system crosstalk. Early in infection, H. pylori activates the PI3K-AKT-β-catenin signaling axis in gastric epithelial cells, inducing NGF overexpression and promoting sensory nerve axon hyperplasia alongside TAN. Furthermore, CagA enhances ROS resistance in gastric cancer stem cells, suppresses autophagy, and promotes the survival of malignant clones (73).

Transient receptor potential (TRP) channels, which are widely distributed in the nervous system and function as pain receptors, are modulated by the gut microbiota, including H. pylori, to influence macrophage differentiation. Following H. pylori infection, TRPM2-deficient macrophages exhibit M1 polarization, which intensifies gastric inflammatory responses. In contrast, TRPM7 promotes M2 macrophage polarization, inhibiting apoptosis and enhancing tumor-promoting functions (74, 75). Through “reverse signaling,” H. pylori also activates the NF-κB pathway in dendritic cells via the TLR4/MyD88 axis, inducing PD-L1 expression and inhibiting CD8+ T cell function. Tregs, meanwhile, inhibit neuronal apoptosis by secreting IL-35, thereby maintaining the density of tumorigenic nerve fibers (76, 77). Another study revealed an enrichment of mucus-degrading bacteria, specifically Akkermansia spp. and Ruminococcus spp., at H. pylori infection sites. This enrichment, coupled with a significant reduction in goblet cells, constitutes a dual disruption that elevates cancer risk and exacerbates deterioration of the intestinal environment (78). In addition to its impact on the gastric microbiota, H. pylori also exerts certain effects on the colonic microbiota. This may be related to the impairment of the gastric barrier and symptoms of gastric hypochlorhydria. The weakening of the acidic environment drives a large number of bacteria to escape from the gastric environment and migrate to the colon. Furthermore, experiments have found that H. pylori can induce a significant proliferation of antigen-specific Th17 cells in the small intestinal and colonic mucosa, providing favorable regulation for the survival of pro-tumorigenic microbiota, which is closely associated with poorer prognosis in CRC patients (79). In summary, H. pylori infection mobilizes the neuro-immune system through various mechanisms and reshapes the gut microbiota. It induces pro-inflammatory cytokines or metabolites that damage the intestinal barrier, thereby triggering a pro-carcinogenic microenvironment.

#### 3.2.2 Fusobacterium nucleatum

Fusobacterium nucleatum, a prominent tumor-promoting bacterium enriched in gastrointestinal cancers, has undergone numerous experimental investigations focusing on its mechanistic link with CRC (80, 81). Studies have shown that Fn can suppress METTL3’s m6A modification, promoting cancer metastasis (82). Fn also activates the TLR4/Myd88/NF-κB signaling pathway, transcriptionally upregulating miRNA-155-5p, which negatively regulates the expression of the DNA mismatch repair protein MSH6 (83, 84). This leads to increased genomic instability and impairs mismatch repair capability, ultimately promoting tumor cell immunogenic escape. Fn’s specific adhesin, FadA, binds to E-cadherin’s extracellular domain, activating the β-catenin signaling pathway and driving CRC cell proliferation and the release of inflammatory factors such as IL-8 and TNF-α (85). Additionally, Fn-derived LPS activates colon epithelial cell CCL2 secretion through TLR4 signaling, recruiting monocytes that differentiate into pro-inflammatory macrophages, which release IL-1β, inducing Th17 cell differentiation and IL-17A/F production (86, 87).

The IL-17 family of cytokines drives tumorigenesis in the immune system and enhances CAF IL-6 secretion indirectly regulated by IL-23 (60, 88, 89). This occurs through the activation of sensory neuron TRPV1 channels and the promotion of CGRP release, which has been shown to promote gastrointestinal tumor progression (12). Furthermore, LPS promotes BDNF secretion, enhancing tumor cell stemness and metastasis via TrkB (90, 91). The role of BDNF in promoting tumorigenic neurogenesis in gastrointestinal cancers has been previously discussed (92).

Moreover, short-chain fatty acids (SCFAs) secreted by Fn can regulate Th17 cell responses by activating the FFAR2 receptor on intestinal chromaffin cells (93, 94). The formic acid produced by Fn can also specifically promote the expansion of Th17 cells in an AhR signaling-dependent manner, thereby exacerbating intestinal barrier dysfunction and promoting the development of gastrointestinal tumors (95). Beyond mechanistic studies of Fn and CRC, Sen et al. identified iron overload as a significant factor contributing to CRC’s poor prognosis. Iron overload enhances the secretion of tumor-promoting chemokines such as CCL8 by inhibiting inhibitory phosphorylation of NF-κB p65 in macrophages, while TLR4/NF-κB signaling can be suppressed under iron deficiency conditions (96).

### 3.3 Metabolite-mediated rewiring of tumor physiology

#### 3.3.1 Modulating neurotransmission

The gut microbiota directly influences neuronal excitability and synaptic plasticity by producing, consuming, or inducing key neuroactive molecules, thereby shaping a tumor microenvironment that can either promote or suppress tumor growth. In colorectal cancer stem cells, Zhu et al. demonstrated that the bacterial metabolite isovalerate (IVA) activates intestinal serotonergic neurons by inhibiting the chromatin-binding ability of the NuRD complex, which lifts its transcriptional repression of the tryptophan hydroxylase-2 (Tph2) gene promoter (97, 98). This process elevates 5-HT synthesis, and intestinal 5-HT subsequently activates the Wnt/β-catenin signaling pathway via its receptors to drive colorectal tumor growth and invasion. Furthermore, 5-HT receptor 5-Hydroxytryptamine receptor 2B (HTR2B) expression is significantly upregulated in gastric adenocarcinoma tissues, where it correlates positively with poor patient prognosis (99). Mechanistically, activated HTR2B complexes with the tyrosine kinase Fyn to directly phosphorylate the PI3K regulatory subunit p85, triggering the Akt/mTOR signaling cascade. This pathway remodels tumor cell metabolism by upregulating hypoxia-inducible factor HIF1α and the lipid transporter ABCD1, thereby inhibiting lipid peroxidation and ferroptosis (100).

Additionally, IL-33 can induce 5-HT secretion via the PLC-γ1-TRPA1 signaling pathway in neuroendocrine channels, offering a new perspective on intestinal homeostasis disorders (101). Through its receptors HTR2A and HTR3A, 5-HT activates prostaglandin E2 (PGE2) synthase (PTGES) in macrophages to promote PGE2 release. PGE2 then acts directly on intestinal stem cells (ISCs) via its receptors EP1 and EP4 to maintain their self-renewal and pluripotency (102). A separate study, however, reveals a distinct immunomodulatory mechanism for 5-HT, whereby tumor-infiltrating T cells employ TGM2 to “hijack” 5-HT molecules and convert them into anti-tumor weapons. In CD8+ T cells, 5-HT enhances glycolytic metabolism via TGM2-mediated GAPDH 5-HTylation, which supports CD8+ T cell proliferation, activation, and effector function by increasing ATP production and intermediate metabolite accumulation (103).

The enzyme TGM2 thus decouples the tumor-promoting and anti-tumor effects of 5-HT. While 5-HT drives malignancy in tumor cells through classical receptor signaling, TGM2-mediated GAPDH 5-HTylation reprograms glycolytic metabolism in CD8+ T cells to establish a “metabolic checkpoint,” transforming 5-HT from a tumor-promoting factor into an immunostimulant. This duality indicates that the biological effects of 5-HT depend critically on its cellular target and molecular modification mechanisms. Targeting TGM2 activation or HTR2B inhibition may therefore hold clinical translational significance. Currently, 5-HT_3_ receptor antagonists such as ondansetron and granisetron are used to manage acute nausea and vomiting induced by radiotherapy and chemotherapy, while 5-HT_4_ receptor agonists like mosapride treat gastrointestinal motility disorders (104). Nevertheless, the precise mechanisms of 5-HT in different cellular subpopulations within the tumor microenvironment remain unclear (105), and further detailed studies are required to determine whether its expression levels differ in metastatic lesions.

As a classical inhibitory neurotransmitter, Gamma-Aminobutyric Acid (GABA) is secreted not only by neurons but also originates from the intestinal flora, where it and its receptor subunits play crucial roles in gastrointestinal tumors. Research demonstrates that microbially derived GABA interacts with host GABA receptor signaling to synergistically regulate colon tumorigenesis (106). Regarding anticancer mechanisms, early work revealed that intestinal flora-derived GABA significantly inhibits colorectal cancer cell proliferation *in vitro (*107). Mechanistic studies indicate that intestinal GABAergic neurons secrete FMRFamide-like neuropeptides (FLP-6) to specifically suppress the transcription factors ZIP-10 and KLF-1, thereby activating the PMK-1/p38 MAPK signaling cascade (108).

GABA signaling directly influences ILC3 function. Recent work demonstrates that GABA markedly decreases insulin-like growth factor binding protein 7 (IGFBP7) transcription by downregulating expression of the LIP isoform of the transcription factor C/EBP-β. Acting as an autocrine signal, IGFBP7 then binds the IGF1 receptor (IGF1R) on ILC3s, thereby inhibiting their proliferation and limiting IL-17A production (51).

At the level of cancer-promoting mechanisms, studies such as those by Wei have identified abnormally high expression of the δ subunit GABA receptor (GABRD) in gastric cancer tissues. Systematic screening of downstream effectors confirmed that GABRD stabilizes cyclin D1 (CCND1) protein by inhibiting its ubiquitin-mediated degradation, thereby driving cell cycle progression and blocking p53-dependent apoptosis (109). Conversely, the GABA_B receptor subunit GABABR1 is expressed at low levels in colorectal cancer. Wang’s team found that its loss induces epithelial-mesenchymal transition (EMT) and enhances tumor cell migration and invasion by activating the Hippo/YAP1 signaling axis (110).

Inadequate sleep also influences tumorigenesis. Sleep deprivation elevates peripheral blood GABA levels, which promotes miR-223-3p expression in colon cancer cells. Through exosomes, miR-223-3p induces macrophage M2 polarization and IL-17 secretion to stimulate tumor cell proliferation and migration. Furthermore, miR-223-3p stabilizes cMYC and drives colon cancer metastasis by suppressing the E3 ubiquitin ligase CBLB, thereby reducing cMYC protein degradation (107). In summary, GABA acts as a concentration-dependent bidirectional regulator with complex biological effects in the gastrointestinal tumor microenvironment. It influences tumor progression through multiple mechanisms, including selective receptor subunit activation, rhythmic secretion, and epigenetic modification, underscoring its potential value as a therapeutic target. Future research should further analyze the spatiotemporal dynamics of the GABA signaling network and its interaction with tumor metabolic reprogramming to provide a theoretical foundation for developing precision therapies.

#### 3.3.2 Shaping immunometabolism

Beyond directly binding to receptors, microbial metabolites can also serve as metabolic substrates for epigenetic modifiers, fundamentally reprogramming the function and fate of immune cells within the tumor microenvironment. Research also found that specific gut microbiota regulate colonic sympathetic ganglia (CG-SMG) expression by producing SCFAs. This suppresses sympathetic nerve activation, and vagal afferent neurons sense microbiota metabolite changes, regulating sympathetic nerve activity. SCFAs activate G protein-coupled receptors, inhibiting NF-κB nuclear translocation and promoting cancer cell apoptosis (111). Butyrate, a specific SCFA, interacts with the GPR109A/HOPX axis to enhance the memory and cytotoxicity of CD8+ T cells, thereby improving the tumor immune microenvironment (112). *In vitro* analyses reveal a significant reduction in butyrate-producing bacteria in the microbiota of gastric cancer patients, and mouse model studies demonstrate that butyrate suppresses immunosuppressive factor production by downregulating STAT3 and NF-κB (113). Another study using a mouse model of bacterial depletion showed that SCFAs promote the recovery of intestinal neurons and participate in neurogenesis, indicating that an intact intestinal microbiota supports intestinal nerve generation (67). Consequently, microbiota dysbiosis can lower SCFA levels, which activates colonic sympathetic ganglia neurons, increases catecholamine release, inhibits gastrointestinal motility, and promotes tumor progression.

Tryptophan metabolites help maintain intestinal homeostasis through three classical pathways: the kynurenine (Kyn), indole derivative, and serotonin pathways (114). These three pathways have been extensively explored, with microorganisms playing a significant role (115). Tryptophan-metabolizing bacteria, such as Bacteroides and Lactobacillus, degrade tryptophan to produce metabolites like kynurenine, activating the aryl hydrocarbon receptor (AhR) and driving the formation of an immunosuppressive TME (116). A study found that the deubiquitinating enzyme USP14 enhances tryptophan metabolism and inhibits cytotoxic T-cell function by stabilizing the indoleamine 2,3-dioxygenase 1 (IDO1) protein (117). As IDO1 is an immunosuppressive enzyme, inhibiting USP14 in a colorectal cancer model reduced IDO1 expression, reversed T-cell exhaustion, and enhanced anti-PD-1 therapy efficacy without activating AhR (118). In this pathway, Fang et al. experimented with indole-3-carboxylic acid (ICA), a derivative of the probiotic Lactobacillus gallinarum. Their research revealed that ICA can downregulate IDO1 by competing with the aryl hydrocarbon receptor (AHR), reverse PD-1 efficacy, and reshape the tumor microenvironment (119). Another probiotic, Bifidobacterium breve, secretes indole-3-lactic acid (ILA). A recent experiment found that ILA inhibits the proinflammatory phenotype of macrophages through the AhR/AKT pathway, antagonizing colitis-associated cancer (120). While indole-3-lactic acid was previously known to inhibit IL-17 signaling to antagonize tumors, the protective effects in this mouse model were abolished by an AhR antagonist, confirming that bacterial metabolites directly regulate immune homeostasis through AhR (121). This has led to the discovery of various potential methods to promote cancer treatment through microbiota (122, 123). A previous study combined ginseng polysaccharides (GPs) with αPD-1 monoclonal antibodies, enhancing microbiota sensitivity and improving probiotic distribution (124). Wang et al. discovered that a diet rich in anthocyanins and dietary fiber can alter gastrointestinal microbiota distribution, enriching Bacteroides uniformis and Lactobacillus, thereby promoting ILA secretion. This potential high value of black rice diet may provide a new perspective for preventing intestinal tumors (125).

### 3.4 Microbial interactions with systemic neuroimmune reflexes

The influence of the gut microbiota on tumor progression extends beyond the local microenvironment to exert potent distal control over distant tumors via systemic neuroimmune pathways. These evolutionarily conserved reflex arcs, integrating the central nervous system, peripheral nerves, and distal immune organs, facilitate the rapid coordination of systemic immunity. Evidence indicates that the microbiota and their metabolites are central regulators of these pathways, shaping the host’s overall anti-tumor immune status.

The vagus nerve bridges peripheral immunity and the brain, extensively innervating the intestinal wall. A well-defined neuroimmune reflex, the cholinergic anti-inflammatory pathway (CAP), involves vagal afferent fibers sensing peripheral inflammatory signals and relaying them to the brainstem to ultimately suppress pro-inflammatory cytokine release, such as TNF-α, and systemically dampen inflammation (126, 127). Vagally released acetylcholine can also promote cancer cell metastasis through the AMPK/MACC1 signaling pathway, indicating direct relevance of microbiota-vagus communication for cancer (128).

As a key neuroimmune hub, the spleen is under precise regulatory control. Wang et al. demonstrated that splenic memory CD4^+^ T cells specifically secrete trefoil factor 2 (TFF2) upon vagus nerve stimulation or adrenergic signaling, a process regulated by the inflammatory reflex pathway (129). TFF2 binds the CXCR4 receptor on MDSCs, thereby blocking MDSC-mediated suppression of T cell function via PD-L1, ARG1, and ROS. The anti-tumor effect of TFF2 is notably neuro-dependent, as vagotomy abolished DSS-induced TFF2 release and exacerbated MDSC accumulation and tumor progression in a colorectal cancer model. Bone marrow transplantation confirmed that hematopoietic-derived TFF2 primarily drives MDSC suppression, and exogenous TFF2 significantly reduced MDSCs while enhancing CD8^+^ T cell tumor-killing activity (55). These findings highlight the spleen’s role as a neuroimmune interface, dynamically regulating MDSC generation and function via the vagus-norepinephrine-TFF2 axis to maintain anti-tumor immunity. As fundamental modulators of gut neural signaling, the microbiota constitute an upstream component of this axis. Chang et al. identified a stress circuit wherein the brain stimulates vagal fibers targeting the duodenal submucosal glands under stress, leading to splenic dilation that may reverse stress-induced reductions in intestinal inflammation (130).

The gut microbiota provides a crucial biological basis for the influence of psychological stress and negative emotions on tumor progression, a process that essentially constitutes remote regulation achieved through systemic neuro-immune reflexes. Negative emotions can promote intestinal inflammation and are significantly associated with gastrointestinal tumor progression. Psychological stress damages oxytocin (Oxt) neurons in the hypothalamus, and their depletion accelerates colorectal cancer progression. Conversely, activating hypothalamic Oxt neurons inhibits tumor growth, an effect reversible by resection of the celiac ganglion–superior mesenteric ganglion (CG–SMG), underscoring the mediating role of neural pathways (131). Functioning as a core component of systemic reflexes, the gut microbiota further amplifies these effects through reverse signaling: studies in germ-free mice show that the absence of a normal microbiota leads to underdevelopment of the blood-brain barrier, dysregulation of the hypothalamic-pituitary-adrenal (HPA) axis, and reduced BDNF expression. In mature hosts, gut dysbiosis can compromise intestinal barrier integrity, permitting systemic influx of gut-derived damage-associated molecular patterns (DAMPs) and inflammatory mediators that overactivate the HPA axis and sympathetic nervous system, thereby establishing a vicious cycle (132). Conversely, stress-triggered systemic reflexes also regulate anti-tumor immunity via neuro-immune reflex arcs and induce significant changes in gut microbiota composition. Anxiety and stress can promote intestinal inflammation, and under the further influence of negative emotions, may potentially induce cancer, although the specific mechanisms are still under investigation. Under stress conditions, the abundance of gut microbiota changes significantly, with increases in Muribaculum and Alistipes, and a decrease in Akkermansia muciniphila. A. muciniphila has been shown to be crucial for maintaining the intestinal barrier. Given the positive correlation between A. muciniphila and butyrate, research has found that butyrate can downregulate LRP5 and inhibit the activation of the WNT/β-catenin pathway, thereby delaying cancer progression. Piezo1 is a protein widely expressed in cholinergic enteric neurons. Xie et al. found that cholinergic enteric neurons can sense intestinal pressure via Piezo1, thereby regulating intestinal homeostasis. It works together with Piezo2 in peripheral sensory neurons to maintain the intestinal microenvironment (133, 134).

## 4 Spatiotemporal specificity of the gastrointestinal tract

### 4.1 Spatial specificity

The gastrointestinal tract is a critical region under extensive neural regulation and exhibits pronounced regional specificity. Spatially, the stomach receives dual control from the vagus and sympathetic nerves, an innervation pattern that dictates its fundamental roles in digestion and its characteristic responses to stimuli (135). The stomach’s unique microenvironment further shapes its resident microbiota, with Helicobacter pylori being predominant. In contrast, the small intestine and colon possess a more autonomous and integrated enteric nervous system (ENS) (136). Within the colon, tumor incidence is higher in the proximal segment compared to the distal segment (137, 138). Studies indicate that differences in nerve fiber types, density, and neurotransmitter expression between these regions may influence epithelial cell proliferation, apoptosis, and susceptibility to tumorigenesis. Sun et al. analyzed aryl hydrocarbon receptor (AHR) expression in intrinsic sensory neurons (iENs) of the small intestine and colon, finding high AHR levels in the colon. In the ileum, AHR suppresses iEN activation to promote ILC3 proliferation and maintain the intestinal barrier, whereas in the colon it promotes iEN activation to regulate motility (139). The rectum, innervated primarily by the pelvic and hypogastric nerves, is densely populated with sensory nerve endings. Patients with rectal cancer often have lower survival rates than those with colon cancer, a disparity research suggests may be linked to the richer autonomic nerve plexuses surrounding the rectum (140). These plexuses could provide enhanced nutritional support and metastatic pathways for tumor cells, while neuron-derived neurotransmitters and growth factors may also modulate tumor cell behavior (141).

### 4.2 Temporal specificity

Temporally, the gut microbiota and its metabolites are known to be regulated by circadian rhythms (142). The suprachiasmatic nucleus, the central circadian pacemaker, coordinates these rhythms with the hypothalamus (143). This regulation drives significant diurnal fluctuations in microbial abundance, diversity, and metabolic activity. Liu et al., using a mouse model of circadian disruption, identified taurocholic acid (TCA) as a link between the central clock and the tumor microenvironment; TCA can target promoters such as H3K4me1 to promote MDSCs accumulation and tumor progression (144). In the stomach, Helicobacter pylori influences acid secretion and can upregulate BMAL1 expression during circadian disruption. BMAL1 regulates core genes involved in cancer cell growth and invasion, demonstrating complex mechanisms within the gastrointestinal tract (145). For instance, BMAL1 overexpression can target the c-Myc gene to promote colorectal cancer invasion (146).

However, a subsequent study found that BMAL1 knockout in a mouse model promoted cancer metastasis. Target gene screening revealed that BMAL1 can inhibit plasmin production, which induces tumor fibrosis and thereby suppresses the release of tumor-promoting factors (147, 148).

This circadian regulation is bidirectional. Microbiota such as Lactobacillus secrete tryptophan metabolites that play dual roles in different microenvironments, mediating interactions between the nervous and intestinal systems (149). The melatonin precursor 5-HT exhibits marked diurnal variation, and its synthesis is regulated by CLOCK/BMAL1 (150). Metabolites like SCFAs accumulate during the day, enhancing T-cell cytotoxicity in the morning and inhibiting intestinal epithelial diurnal oscillations through histone deacetylase (HDAC) inhibition (151). Different microorganisms and their metabolites follow distinct circadian rhythms, acting individually or collectively on the intestine, though their precise mechanisms require further investigation.

In summary, spatiotemporal specificity is a defining feature of the gastrointestinal tract, forming a multi-level dynamic network during cancer formation and progression. This network represents a potential therapeutic target for gastrointestinal tumors, necessitating intervention strategies that account for spatiotemporal dimensions. Further mechanistic exploration and clinical studies are needed.

## 5 Clinical research and future challenges

Significant advances have been made in the clinical translation of gastrointestinal cancer therapies, driven by synergistic drug mechanisms and novel delivery technologies. Statins demonstrate potent anti-tumor effects by activating ROS generation to induce mitochondrial damage and upregulating indole-3-lactic acid (ILA) secretion to remodel the tumor metabolic microenvironment (120). Chloroquine (CQ) augments the mitochondrial targeting of statins by inhibiting the autophagolysosome pathway, a synergy that substantially improves immunotherapy outcomes (152). For precision delivery, Liu’s team developed a neuropilin-1 (NRP-1)-targeted, responsive liposome that enables spatiotemporally controlled drug release. Legumain protease-mediated cleavage of the carrier backbone triggers the synchronous release of anti-PD-L1 antibodies, which markedly enhances CD8+ T cell infiltration and counteracts regulatory T cell (Treg)-mediated immunosuppression (153). This system was validated in a colorectal cancer PDX model, where it significantly increased tumor regression rates.

Innovations in gut microbiota modulation have created new avenues for treating advanced cancers. A clinical trial by Wong et al. confirmed that combining the MET4 microbiota preparation with PD-1 inhibitors yields promising efficacy. Mechanistic analyses indicate that increased gut abundance of MET4 microbiota in high responders correlates positively with elevated circulating IgG levels, alongside expansion of peripheral blood B cells and a reduced proportion of CD4+ and CD16+ monocytes (154). These results imply that reprogramming the microbiota-immune network may potentiate the effect of immune checkpoint inhibitors (**Table 2**).

**Table 2:** Recent clinical trials targeting nerves and microbiota.

NCT number	Phase	Tumor type	Treatment method	Results	Status
NCT03341143	II	Melanoma	FMT + Pembrolizumab	40% of patients achieved clinical benefit; intestinal microbiota altered; CD8+ T cell activation increased	Active, not recruiting
ChiCTR2100046768	II	MSS Metastatic Colorectal Cancer	FMT + Tislelizumab + Fruquintinib	Median PFS 9.6 months, ORR 20%, DCR 95%; well-tolerated	–
DRKS00014054	II	Pancreatic Cancer	Propranolol + Etodolac	No safety concerns; treatment group: median DFS 16.36 months, recurrence rate 11.1%	Terminated Early
NCT05690048	II	Advanced Hepatocellular Carcinoma	FMT + Atezolizumab/Bevacizumab (A/B)	Not yet reported	Recruiting
NCT06405113	II	Advanced Gastric Cancer	FMT + SOX + Sintilimab	Not yet reported (mouse study showed slowed tumor growth, increased intestinal microbiota diversity, and enhanced immune cell infiltration in SDC+PD1+FMT group)	Recruiting
NCT03245554	I	Gastric Cancer	Propranolol vs Placebo	Propranolol inhibited gastric cancer growth (reduced Ki-67 expression) but did not activate CD8+ T cells; 1-week preoperative administration did not alter AKT/ERK phosphorylation	Not yet recruiting
NCT04130763	I	Gastrointestinal Cancer	FMT Capsules + Anti-PD-1 Therapy	Efficacy data not clearly published	Completed
NCT03772899	I	Melanoma	FMT (Healthy Donor) + Anti-PD-1 Therapy	ORR 65% (20% CR, 45% PR); median PFS not reached at median follow-up of 20.7 months; 16 patients survived	Active, not recruiting

However, this field still faces multiple challenges: 1) The off-target effects of neuromodulatory drugs need to be urgently addressed. For example, although the β-blocker propranolol can inhibit sympathetic nerve-driven tumor metastasis, its cardiovascular side effects lead to dose-limiting toxicity; 2) Spatial heterogeneity in gastrointestinal anatomical sites affects treatment response. Significant differences exist in cholinergic innervation density, microbiota colonization characteristics, and local immune microenvironments between esophageal and colonic tumors, requiring the development of site-specific targeted carriers; 3) There are significant variations in individual gut microbiota composition, making it difficult to establish universal microbiota regulation strategies and avoid treatment failure due to niche competition; 4) The specific mechanisms underlying the circadian rhythm of gut microbiota metabolites and the biological clock remain unclear, posing a challenge in utilizing circadian rhythm to better develop targeted treatment regimens.

## 6 Conclusion

In summary, the neuroimmune network within the gastrointestinal tumor microenvironment constitutes a bidirectional regulatory circuit: neural signals can bypass conventional immune checkpoints to directly promote tumor growth, while immune cells initiate counter-regulatory programs that may either amplify or suppress tumor progression. The gut microbiota acts as a third modulator, converting dietary and environmental cues into bioactive metabolites that can reprogram both neural output and immune responses, thereby shifting the balance toward malignancy or homeostasis. In-depth investigation of this tripartite interaction provides a mechanistic framework for precise intervention in gastrointestinal tumors. Future research should build upon the spatiotemporal specificity of the gastrointestinal tumor microenvironment to comprehensively elucidate the interconnected cascades among these three components, with the goal of achieving durable therapeutic responses in gastrointestinal cancers.

## Glossary

Ach: acetylcholine
A2AR: adenosine A2A receptor
A2BR: adenosine A2B receptor
ADO: Adenosine
ADRBs: β-adrenergic receptors
AhR: Aryl hydrocarbon receptor
ANS: autonomic nervous system
bFGF: basic fibroblast growth factor
BDNF: brain-derived neurotrophic factor
CAFs: cancer-associated fibroblasts
CGRP: calcitonin gene-related peptide
CNS: central nervous system
CRC: colorectal cancer
CREB: cAMP-response element binding protein
DAMPs: damage-associated molecular patterns
DCC: deleted in colorectal carcinoma
EGCs: Enteric glial cells
EMT: epithelial-mesenchymal transition
ENS: enteric nervous system
GABA: Gamma-Aminobutyric Acid
GDNF: glial cell-derived neurotrophic factor
HDAC: histone deacetylase
HIF-1α: hypoxia-inducible factor 1α
Hp: Helicobacter pylori
HTR2B: 5-Hydroxytryptamine receptor 2B
IDO1: indoleamine 2,3-dioxygenase 1
IL-10: interleukin-10
ILC3: type 3 innate lymphoid cells
Kyn: kynurenine
L-DOPA: Levodopa
LPS: Lipopolysaccharide
MDSCs: myeloid-derived suppressor cells
MMP9: matrix metalloproteinase 9
NE: norepinephrine
NGF: nerve growth factor
NRP1: neuropilin-1
PD-1: programmed cell death protein 1
PNI: Perineural invasion
PNS: peripheral nervous system
SCFAs: short-chain fatty acids
STAT3: signal transducer and activator of transcription 3
TAMs: tumor-associated macrophages
TAN: tumor-associated neurogenesis
TCA: taurocholic acid
TFF2: trefoil factor 2
TGF-β: transforming growth factor Beta
Tph2: tryptophan hydroxylase-2
TME: tumor microenvironment
TRPV1: transient receptor potential vanilloid 1
VIP: vasoactive intestinal peptide
VIPR2: vasoactive intestinal peptide receptor 2
β2-AR: Beta-2 adrenergic receptor
5-HT: 5-Hydroxytryptamine.
